# Surveillance of antimicrobial resistance in human health in Tanzania: 2016–2021

**DOI:** 10.4102/ajlm.v12i1.2053

**Published:** 2023-05-22

**Authors:** Neema Camara, Nyambura Moremi, Janneth Mghamba, Eliudi Eliakimu, Edwin Shumba, Pascale Ondoa, Beverly Egyir

**Affiliations:** 1 Epidemiology and Disease Control Section, Ministry of Health, Dodoma, United Republic of Tanzania; 2 Department of Bacteriology, National Public Health Laboratory, Dar es Salaam, United Republic of Tanzania; 3 Health Quality Assurance Unit, Ministry of Health, Dodoma, United Republic of Tanzania; 4 African Society for Laboratory Medicine, Addis Ababa, Ethiopia; 5 Department of Bacteriology, Noguchi Memorial Institute for Medical Research, College of Health Sciences, University of Ghana, Legon, Ghana

**Keywords:** surveillance, antimicrobial resistance, COVID-19, One Health, Tanzania, Africa

## Abstract

**Background:**

Antimicrobial resistance (AMR) surveillance plays an important role in early detection of resistant strains of pathogens and informs treatments decisions at local, regional and national levels. In 2017, Tanzania developed a One Health AMR Surveillance Framework to guide establishment of AMR surveillance systems in the human and animal sectors.

**Aim:**

We reviewed AMR surveillance studies in Tanzania to document progress towards establishing an AMR surveillance system and determine effective strengthening strategies.

**Methods:**

We conducted a literature review on AMR studies conducted in Tanzania by searching Google Scholar, PubMed, and the websites of the Tanzania Ministry of Health and the World Health Organization for articles written in English and published from January 2012 to March 2021 using relevant search terms. Additionally, we reviewed applicable guidelines, plans, and reports from the Tanzanian Ministry of Health.

**Results:**

We reviewed 10 articles on AMR in Tanzania, where studies were conducted at hospitals in seven of Tanzania’s 26 regions between 2012 and 2019. Nine AMR sentinel sites had been established, and there was suitable and clear coordination under ‘One Health’. However, sharing of surveillance data between sectors had yet to be strengthened. Most studies documented high resistance rates of Gram-negative bacteria to third-generation cephalosporins. There were few laboratory staff who were well trained on AMR.

**Conclusion:**

Important progress has been made in establishing a useful, reliable AMR surveillance system. Challenges include a need to develop, implement and build investment case studies for the sustainability of AMR surveillance in Tanzania and ensure proper use of third-generation cephalosporins.

**What this study adds:**

This article adds to the knowledge base of AMR trends in Tanzania and progress made in the implementation of AMR surveillance in human health sector as a contribution to the global AMR initiatives to reduce AMR burden worldwide. It has highlighted key gaps that need policy and implementation level attention.

## Introduction

Antimicrobial resistance (AMR) is a global public health threat with extensive social, health and economic impacts.^1,2^ Globally, it accounts for about 700 000 deaths annually.^3^ Antimicrobial resistance threatens the lives of 10 million people and an economic loss of up to $100 trillion (United States dollar [USD]) per year by 2050, if there are no effective interventions.^3^ It is estimated that the magnitude of the AMR burden falls on low- and middle-income countries.^3^ Antimicrobials are the mainstay of modern medicine; without them, medical procedures, including surgeries, joint replacements, and treatments, such as cancer chemotherapy, could become too risky to be undertaken as healing would take a long time and become costly.^3^

In 2015, the World Health Assembly, through its 68th session, adopted the Global Action Plan on AMR to ensure the treatment and prevention of infectious diseases with quality-assured, safe, and effective medicines available.^4^ The Global Action Plan outlines five strategic objectives, which are: (1) to improve awareness and understanding of AMR; (2) to strengthen knowledge through surveillance and research; (3) to reduce the incidence of infection; (4) to optimise the use of antimicrobial agents; and (5) to ensure sustainable investment in countering AMR.^4^ To support the implementation of the Global Action Plan, during the same year, the World Health Organization (WHO) launched the Global AMR Surveillance System (GLASS), the first global collaborative effort to standardise AMR surveillance.^5^ The GLASS provides a standardised approach for collecting, analysing, and sharing AMR data and documents the status of existing or newly developed national AMR surveillance systems.^5^

In 2016, Tanzania developed the National Action Plan for AMR (2017–2022) following the WHO and Global Health Security Agenda Joint External Evaluation recommendation. Subsequently, a holistic One Health AMR Surveillance Framework was developed to guide the establishment of AMR surveillance systems in the human, animal and environmental health sectors. The country is bordered by more than eight countries, which poses a high risk of pathogen importation into the country. In addition, several socioeconomic, demographic and environmental factors also facilitate the emergence and spread of microorganisms; thus, robust health systems are paramount for detecting, responding, and mitigating the effects of the resistant microbes.

In this current global coronavirus disease 2019 (COVID-19) pandemic situation, where scientists are struggling to find an effective treatment for COVID-19, antibiotics have been widely used to manage COVID-19, either to treat COVID-19 itself or co-infections.^6^ In fact, recent studies have shown the rampant use of antibiotics by most COVID-19 patients without bacterial co-infections.^7^

This paper reviewed AMR surveillance studies and documents the progress made in establishing the AMR surveillance system in the human health sector in Tanzania and provides recommendations for strengthening it. The literature review was essential to contextualise the AMR situation in the past decade and the need to strengthen AMR surveillance.

## Methods

### Data collection

We searched Google Scholar, PubMed, and websites of the Tanzania Ministry of Health and WHO written in English and published from January 2012 to March 2021. We used the search terms: ‘antimicrobial resistance’, ‘bacterial resistance’, ‘antibiotic resistance’; ‘AMR surveillance’, or ‘surveillance’ or ‘cross-section’ or ‘review’; and ‘Tanzania’. All words were searched together, and, in some instances, two of the three words were used. We reviewed guidelines, plans, and reports from the Ministry of Health to describe the Tanzania AMR surveillance system’s objective, surveillance sites, data collection, reporting, analysis, interpretation, and dissemination, coordination of AMR surveillance activities, and funding of AMR surveillance.

### Setting and structure of healthcare system in Tanzania

The United Republic of Tanzania comprises Tanzania’s Mainland and the semiautonomous Islands of Zanzibar, and it lies on the East African coast. The Tanzania 2012 population census was 44 928 923.^8^ Tanzania Mainland has 26 administrative regions, 139 districts and 184 councils. The council divides into divisions, then wards, and streets/villages. The local government authorities (or councils) are the most important administrative and implementation units for public services.

Health services in Tanzania are decentralised into three categories: district (primary level), regional (secondary level), and zonal and national hospital (tertiary level). The district level provides primary health care services through dispensaries at the village level, health centres at the ward level, and Level 1 Hospital at the council level.^9^ Dispensaries provide preventive and curative out-patient services. In contrast, health centres admit patients and sometimes provide surgical services. Council hospitals provide healthcare to referred patients and provide medical and basic surgical services. Regional Referral Hospitals provide specialist medical care. Zonal and national hospitals offer advanced (super specialist) medical care and are teaching hospitals for medical, paramedical, and nursing training.^9^ Public, private and faith-based organisations health facilities, private pharmacies, and accredited drug dispensing outlets provide pharmaceutical services.^10,11^

## Results

### Antimicrobial resistance trends of priority pathogens in Tanzania

**FIGURE 1:**
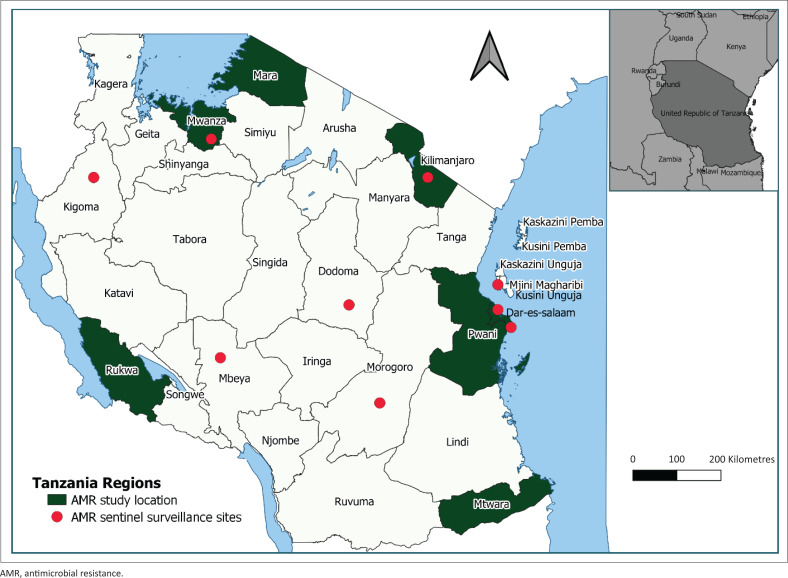
Antimicrobial resistance study locations and sentinel surveillance sites, August 2021, Tanzania.

A total of 10 articles on AMR in Tanzania were retrieved and reviewed. These studies were conducted at either the zonal or regional referral hospitals between 2012 and 2019. Four of the 10 studies were conducted at Kilimanjaro Christian Medical Centre in the Kilimanjaro region, three at Bugando Medical Centre in Mwanza region, two at Muhimbili National Hospital in Dar es Salaam region, and one study each at Maweni Regional Referral Hospital in Kigoma region, Musoma Regional Referral Hospital in Mara region, Sumbawanga Regional Referral Hospital in Rukwa region, St. Benedict Ndanda Hospital in Mtwara region, Bagamoyo District Hospital in Pwani region, Sekou Toure Regional Referral Hospital, Nyamagana District Hospital, and Sengerema District Designated Hospital in Mwanza region (Figure 1). Blood, pus and wound swabs, and urine were the most common laboratory samples analysed (Table 1). All of the studies performed antimicrobial susceptibility testing (AST) using the disk-diffusion method per the Clinical Laboratory Standards Institute guidelines.^12^ The most frequently isolated microorganisms from blood were *Staphylococcus aureus, Klebsiella pneumoniae* and *Escherichia coli;* and from pus, *Pseudomonas aeruginosa* (Table 1). *S. aureus* resistance to clindamycin ranged between 33.3% to 68.4% and erythromycin between 35.6% to 76.3%, while resistance to cotrimoxazole was 82.6% and ampicillin was 100%.^13,14,15,16^ The studies reported low rates of resistance to cefoxitin (27.3%), tetracycline (34.9%), cotrimoxazole (26.5%) and ceftriaxone (11.1%).^14,17,18^ Prevalence of methicillin-resistant *S. aureus* decreased from 41.2% in 2013 to 9.5% in 2015, but rose to 66.7% in 2018.^14,15^ *K. pneumoniae* was resistant to ampicillin (100%), cotrimoxazole (96.3%), ceftriaxone (95.7%), amoxicillin/clavulanate (94.6%), ceftazidime (90.9%), gentamycin (86.4%) and cefepime (75.6%).^13,16,19^ Compared to other Gram-negatives, *E. coli* was more resistant to ampicillin, amoxicillin-clavulanic acid, gentamycin, tetracycline, ciprofloxacin, amikacin, third-generation cephalosporins (ceftazidime and ceftriaxone) and cefepime.^,13,15,16,19,20^ Notably, *P. aeruginosa* was resistant to cefepime (93.8%).^13^ Overall, most studies documented high resistance rates of Gram-negative bacteria to third-generation cephalosporins.^17,21,22^

**TABLE 1:** Antimicrobial resistance trends of GLASS priority pathogens in Tanzania, 2012–2021.

S/N	Title of the study, Ref	Year of study	Sources of isolates	Study population	Organisms recovered most	Antimicrobial resistance rates (%)	Study type
1	Causative agents and antimicrobial resistance patterns of human skin and soft tissue infections in Bagamoyo, Tanzania.^14^	February to October 2012	Wound swabs	Out-patient	*Staphylococcus aureus*	Penicillin (96.2), tetracyclines (34.9), cotrimoxazole (26.5), Erythromycin (35.6) and clindamycin (33.3)	Hospital-based
2	Antimicrobial resistance pattern: A report of microbiological cultures at a tertiary hospital in Tanzania.^21^	June 2013 to May 2015	Blood	Children and adults	*Staphylococcus aureus*	Methicillin (34.6) MRSA < from 41.2% in 2013 to 9.5% in 2015	Hospital-based
					*Klebsiella pneumoniae*	3rd-generation cephalosporin (38.5)	
					*Salmonella* spp.	3rd-generation cephalosporin (39.0)	
			Urine		*Escherichia coli*	3rd-generation cephalosporin (29.3)	
			Pus swab		Gram-negative bacteria	3rd-generation cephalosporin > from 26.5 in 2014 to 57.9 in 2015	
3	Patterns of infections, aetiological agents and antimicrobial resistance at a tertiary care hospital in northern Tanzania.^17^	August 2013 to August 2015	Stool, sputum, blood, wound/pus swab	In-patients from surgical and medical wards	**Gram-negative bacteria** (*Proteus* spp., *Escherichia coli, Klebsiella* spp. *and Pseudomonas* spp.)	Cefazolin (72.9), ceftriaxone (51.8), ceftazidime (37.4)	Hospital-based
					*Staphylococcus aureus*	Cefoxitin (27.3), Penicillin (100)	
4	Prevalence and antimicrobial resistance patterns of extended spectrum beta-lactamase producing *E. Coli* in human isolates at Kilimanjaro Medical Centre, Moshi, Tanzania.^20^	April 2016 to May 2016	Urine and stool	All patients	*Escherichia coli*	Cephalosporin (70.0), amikacin (60.0)	Hospital-based
5	Deciphering risk factors for blood stream infections, bacteria species and antimicrobial resistance profiles among children under five years of age in North-Western Tanzania: A multicentre study in a cascade of referral health care system.^16^	July 2016 to October 2017	Blood	< 5 years of age	*Klebsiella pneumoniae*	Ampicillin (100.0), trimethoprim-sulfamethoxazole (SXT) (96.3), AMC Amoxycillin-clavulanate (94.6), CAZ Ceftazidime (90.9), CRO Ceftriaxone (95.7)	Hospital-based
					*Staphylococcus aureus*	Ampicillin (100.0), SXT trimethoprim-sulfamethoxazole (82.6), ERY Erythromycin (65.2)	
					*Escherichia coli*	Ampicillin (100.0), SXT trimethoprim-sulfamethoxazole (94.4), ciprofloxacin (52.9), AMC Amoxycillin-clavulanate (94.1), CRO (58.8), CAZ (52.9)	
6	Laboratory confirmed puerperal sepsis in a national referral hospital in Tanzania: Etiological agents and their susceptibility to commonly prescribed antibiotics.^19^	December 2017 to April 2018	Blood and endocervical swabs	Women suspected of puerperal sepsis	*Escherichia coli*	Ceftriaxone (64.7), ampicillin (67.6) and ceftazidime (63.2)	Hospital-based
					*Klebsiella* spp.	Ceftriaxone (77.3), gentamicin (86.4), ampicillin (81.8), ceftazidime (86.4)	
					*Staphylococcus aureus*	Methicillin (53.8)	
7	Antibiotic resistance in aerobic bacterial isolates from infected diabetic foot ulcers in North Eastern Tanzania: An urgent call to establish a hospital antimicrobial stewardship committee.^22^	September 2018 to March 2019	Pus swab	Diabetic foot ulcers admitted at the surgical department	*Escherichia coli*	Ceftriaxone (50.0), Amoxicillin and clavulanic acid (47.6)	Hospital-based
					*Pseudomonas aeruginosa*	Amikacin (42.9)	
8	Antibiotic susceptibility patterns of bacterial isolates from routine clinical specimens from referral hospitals in Tanzania: A prospective hospital-based observational study.^15^	October 2018 to September 2019	Ear pus, urine, wound pus, stool and blood	In-patient and out-patient	*Staphylococcus aureus*	Erythromycin (76.3), Gentamycin (54.0), Ciprofloxacin (40.0) and Clindamycin (34.9). MRSA (66.7)	Hospital-based
					*Escherichia coli*	Ampicillin (100.0), Amoxicillin-Clavulanic Acid (75.0), Gentamicin (70.2), Tetracycline (70.2) and Ciprofloxacin (42.6)	
9	Etiologies of bloodstream infection and antimicrobial resistance: A cross sectional study among patients in a tertiary hospital, Northern Tanzania.^18^	April 2019 to June 2019	Blood	Out-patient and in-patient	*Staphylococcus aureus*	1 out of 3 isolates was resistant to Meropenem, Cefotaxime, Amikacin, Gentamicin, Imipenem, and ceftriaxone (11.1)	Hospital-based
10	The existence of high bacterial resistance to some reserved antibiotics in tertiary hospitals in Tanzania: A call to revisit their use.^13^	July 2019 to November 2019	Blood, urine, pus,	Out-patient and in-patient	*Staphylococcus aureus*	Clindamycin (68.4)	Hospital-based
					*Pseudomonas aeruginosa*	Cefepime (93.8)	
					*Klebsiella* spp.	Cefepime (75.6)	
					*Escherichia coli*	Cefepime (56.3)	
					*Klebsiella* spp.	Ceftriaxone (77.3), gentamicin (86.4), ampicillin (81.8), ceftazidime (86.4)	

MRSA, methicillin-resistant *Staphylococcus aureus*; S/N, serial number; Ref, reference.

### Progress in implementation of antimicrobial resistance surveillance

#### Coordination

In 2018, Tanzania took the first step of developing a One Health National AMR surveillance framework to guide the establishment of AMR surveillance programmes in the country. The framework provides a holistic approach to monitor trends of infections and resistance that will inform standard treatment guidelines that support best practices for patient care, and links AMR information from the human, animal and environmental health sectors.^23^ The objectives of AMR surveillance are to routinely collect, analyse, and interpret quality AMR data to generate evidence on trends and the burden of the WHO priority pathogens, and monitor AMR interventions’ effectiveness. The country also established a national Multi-Sectoral Coordinating Committee (MCC) to oversee and coordinate all AMR-related activities in all sectors. The Chief Medical Officer of the Ministry of Health and the Director of Veterinary Services, Ministry of Livestock and Fisheries alternate as co-chair of the committee. The committee is composed of representatives from the human, animal and environmental health sectors, as well as livestock and food production stakeholders, and those from medical and agricultural universities. The WHO, Food and Agriculture Organization, United States Centres for Disease Control and Prevention, Management Science for Health and World Organization for Animal Health, are also represented in the MCC. There are designated national AMR focal points from animal and human sectors that form part of the MCC secretariat, as well as four multisectoral Technical Working Groups on (1) awareness, effective communication and education; (2) knowledge, surveillance, research and sustainable investments; (3) sanitation, hygiene and infection prevention and control; and (4) antimicrobial use stewardship. The MCC and surveillance TWG meets at least once every quarter of the year. The whole coordination structure operates under the ‘One Health’ and whole-of-government approach.

#### Antimicrobial resistance surveillance system

Tanzania started with laboratory-based AMR surveillance in healthcare settings, as laboratory-based surveillance is the most efficient AMR burden determination method.^24,25^ In the first phase of the national Tanzania AMR surveillance there were two laboratory levels: coordinating laboratory and site (sentinel/participating) laboratories. As of 2022, there are a total of nine AMR sentinel sites which are active and functional. The sentinel sites include Muhimbili National Hospital, Mbeya Zonal Referral Hospital, Bugando Medical Centre, Kilimanjaro Christian Medical Centre, Mnazi Mmoja Hospital in Zanzibar, Temeke Regional Referral Hospital in Dar es Salaam region, Morogoro Regional Referral Hospital in Morogoro region, Maweni Regional Referral Hospital in Kigoma region, and Benjamin Mkapa Hospital in Dodoma region (Figure 1). The National Health Laboratory (NHL) is the national coordinating laboratory, and its primary roles include: developing AMR national standard operating procedures; training, mentoring and supervising sentinel laboratories; conducting external quality assurance and monitoring internal quality assurances done by sentinel laboratories; and compiling, aggregating, analysing, visualising and disseminating national AMR surveillance data to the national MCC and the GLASS. On the other hand, sentinel laboratories isolate and identify organisms; perform susceptibility tests; store isolates as per national standardised operating procedures; produce and share timely antibiograms with clinicians; and conduct internal quality assurances. Antimicrobial resistance surveillance involves the routine collection of blood and urine specimens from in and out-patients with clinical signs and symptoms attending the hospitals. Clinicians decide whether to take samples for microbiological culture based on the patient’s clinical assessment. Presently, the participating laboratories employ phenotypic methods for pathogen identification and disk diffusion for AST. The AST is a laboratory procedure to identify effective antimicrobial agents that kill or prevent the growth of isolated pathogens recovered from samples of individual patients.^26^ Antimicrobial susceptibility testing results guide clinicians and service providers to decide on target therapy, preserve drugs, and evaluate treatment services.^26^ Notably, continuous surveillance for resistance patterns is crucial due to the mutations in bacterial DNA.^26^ The AST is performed and interpreted according to international guidelines such as the Clinical and Laboratory Standards Institute guidelines. The AST results are categorised into either susceptible (S) or non-susceptible, which include intermediate (I) and resistant (R) according to clinical breakpoints defined by Clinical and Laboratory Standards Institute. Patient clinical data, including infection origin (community or hospital), age, gender and admission types (out-patient, in-patient, general ward or intensive care unit) are collected regardless of culture positivity or negativity. Infection origin are categorised as hospital-acquired (specimen from an in-patient admitted for > 2 days) or community-acquired (specimen from an out-patient or in-patient admitted for ≤ 2 days).^23^

Clinical data and AST positive culture results are recorded in the reporting forms and entered into the laboratory information system and the World Health Organization Network (WHONET), a freely available system for capturing, analysing and sharing AMR data in a standardised format. Data import into WHONET can be semi-automated using the add-on Baclink software (WHO Collaborating Centre for Surveillance of Antimicrobial Resistance, Boston, Massachusetts, United States), which allows for import from other data sources, for example, text files exported from a laboratory information management system (LIMS) or directly from a laboratory instrument.^27^ However, WHONET intentionally provides only a solution for basic laboratory specimen management and result reporting and does not have comprehensive LIMS functionality.^27^ Laboratory departments communicate AST results immediately to clinicians as well as the infection prevention and control and AMR teams for appropriate treatment and control programs in the local setting. Target pathogens for monitoring and reporting as per the national and WHO priorities include *E. coli, K. pneumoniae, Acinetobacter baumannii, S. aureus, Neisseria meningitidis, Streptococcus pneumoniae, Salmonella* spp., *Shigella* spp., *Pseudomonas* spp. and *Neisseria gonorrhoeae.*

#### Data analysis, interpretation and dissemination

The participating laboratories must clean, collate, analyse, and create site-specific bacterial antibiograms every month. In addition, annual AMR surveillance reports are shared with the relevant clinical departments and hospital committees to increase hospital and community AMR awareness, inform treatment policies at the health facility, and encourage continued participation in the surveillance system.

**FIGURE 2:**
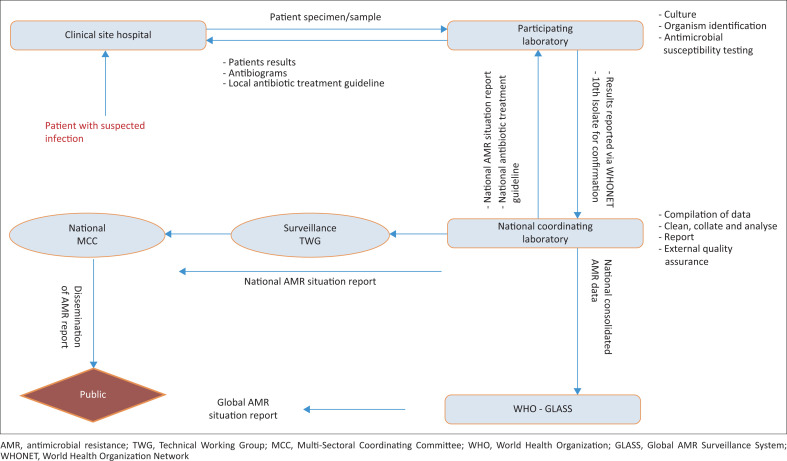
Antimicrobial resistance data flow in Tanzania (9 January 2022).

Antimicrobial resistance data from surveillance sites are centrally stored and managed at the NHL. The NHL conducts data quality checks, analysis, and visualisation, generates official AMR reports, and provides long-term data storage. Antimicrobial resistance surveillance reports, including trends and resistant pathogen prevalences, are generated at least twice a year and disseminated to stakeholders after approval by the AMR Surveillance Technical Working Group and the national MCC. At the same time, the clean AMR data set is transmitted to the GLASS (Figure 2). The AMR surveillance data guides strategies and policies for combating AMR. It also provides opportunities for in-depth scientific research that generates additional knowledge on AMR. However, sharing of surveillance data among sectors is yet to be strengthened, that is, environment, health and animal. Tanzania started reporting AMR data to GLASS in June 2021.

#### Quality assurance and standards

All nine sentinel sites participate in the external quality assurance which is being supported by NHL and African Society for Laboratory Medicine under the project called External Quality Assessment for Africa. External quality assurance is done twice a year for all sites and involves most of the GLASS priority pathogens, including *E. coli, K. pneumoniae, A. baumannii, S. aureus, N. meningitidis, S. pneumoniae* and *Salmonella* spp. As per the AMR surveillance framework, all isolates are to be stored at −70 °C for future studies. Isolates with unusual, unexpected, or indeterminate resistance patterns are sent to the NHL for confirmatory testing and AST. Also, every 10th isolate from each site is sent to NHL for quality assessment.

### Funding

Funds to run the nine AMR surveillance sentinel laboratories are contributed by the Government of the United Republic of Tanzania, the Fleming Fund, and the United States Agency for International Development fund under the Infectious Disease Diagnostic and Surveillance project. Therefore, there is a need to establish a sustainable funding mechanism for AMR activities in Tanzania.

## Discussion

This review has shown that the most frequently isolated microorganisms from blood were *S. aureus, K. pneumoniae* and *E. coli;* from urine, *E. coli*; and from pus, *P. aeruginosa*. Most studies documented high resistance rates of Gram-negative bacteria to third-generation cephalosporins. Importantly, significant progress has been made in establishing AMR surveillance; nine sentinel sites across Tanzania have been established and are generating data and there are suitable and clear coordination structures and platforms for multisectoral engagement and collaboration under One Health. However, sharing of surveillance data between sectors is yet to be strengthened. There are also few laboratory staff well trained on AMR practices.

Studies in Tanzania reveal increasing bacterial resistance to third-generation cephalosporins. In Tanzania, ceftriaxone is reportedly prescribed excessively and inappropriately in hospital settings,^28^ and this may explain the observed third-generation cephalosporins resistance trends. If this trend continues, clinicians will resort to broad-spectrum antibiotics, such as carbapenem, which are the last resort according to the Tanzania treatment guideline.^29,30^ If such a situation occurs, effective, quality, and affordable healthcare provisions, the core fundamentals for universal health coverage, will be far from being realised.

Progress made in establishing the AMR surveillance system in the country is commendable. A total of nine sentinel sites have been established. There are suitable coordination structures and platforms for multisectoral engagement and collaboration under the ‘One Health’.^31^ The AMR sentinel surveillance sites are representative and provide AMR trends and burden data. AMR surveillance system has helped to standardise routine microbiological cultures and AST in hospitals, particularly those participating in AMR surveillance according to global standards (MCC meeting minutes of 11 May 2021, unpublished). However, the facilities still face challenges while implementing AMR surveillance, including a lack of interoperability between the sentinel site laboratory information system and WHONET to enable automatic data transfer between the two systems. The double entry of the same information in two different systems exhausts and overworks the laboratory staff. A lack of fit-for-purpose LIMS and open-source LIMS software with technical standards and functionality for AMR surveillance is a particular concern in low- and middle-income countries.^27^ Although WHONET is a functional and useful repository for microbiological data with capabilities for standardised data sharing, it lacks full LIMS functionality.^27^ Thus, there is an urgent need for investment in laboratory information technology infrastructure and data management systems that can capture high-quality laboratory and patient management data.

There are few well-trained laboratory staff at the sentinel sites for data analysis and the production of antibiograms, which can be shared with the clinicians and AMR teams to inform on the appropriate treatment and measures to tackle AMR at the hospital or community. At the surveillance sites, frequent stock out of AST reagents and analysis, inadequate resources, and poor laboratory infrastructure for phenotypic and genotypic analysis is commonplace. Although AMR surveillance receives much support from the government as per human resources and infrastructure, there are also funds from donors. A sustainability plan is essential to prevent over-dependence on donors over time. These challenges are unique in Tanzania and have also been reported elsewhere in Africa.^32,33^ However, despite the challenges, AMR surveillance is still ongoing in the country.

Sharing of surveillance data between sectors is yet to be strengthened. Antimicrobial resistance is a broad and complex issue affecting the animal, human and environmental sectors; thus, a multisectoral and multidisciplinary combat approach is needed. Antimicrobials are also widely used in animals for treatment and growth promoters. In addition, evidence suggests that antimicrobial use in animals contributes significantly to the development of AMR in humans,^34^ necessitating a comprehensive and coordinated AMR surveillance system that can continuously share AMR data between sectors to inform public health interventions.

This study has some limitations. The review was based on reports only. We did not seek additional inputs and insights from AMR stakeholders through a standardised questionnaire or interview. As such some comprehensive views and perspectives may have been missed out. The AMR trends in Tanzania presented here should be interpreted with caution as the review was only based on 10 surveillance articles.

### Conclusion

Tanzania is currently implementing AMR surveillance in nine hospitals, and reporting of AMR data to GLASS has commenced. There are well-established AMR coordination mechanisms at health facilities and national levels to effectively implement and utilise the AMR information. Although there are challenges affecting implementation, the current AMR surveillance system in place is useful, reliable and capable of better performance. There is a need to develop, implement and build an investment case study for the sustainability of AMR surveillance in Tanzania. We recommend that the government creates a fit-to-purpose laboratory information system with functionality able to link with other systems; develops a mechanism for sustainable financing for laboratory infrastructure development and continuous supply of reagents, commodities, and laboratory materials; and invests hugely in building human capacities for bacterial identification and AST and data analysis, interpretation and utilisation.
